# Quantifying microstructures of earth materials using higher-order spatial correlations and deep generative adversarial networks

**DOI:** 10.1038/s41598-023-28970-w

**Published:** 2023-01-31

**Authors:** Hamed Amiri, Ivan Vasconcelos, Yang Jiao, Pei-En Chen, Oliver Plümper

**Affiliations:** 1 Department of Earth Sciences, Utrecht University, Utrecht, The Netherlands; 2 Materials Science and Engineering, Arizona State University, Tempe, USA; 3 Mechanical and Aerospace Engineering, Arizona State University, Tempe, USA

**Keywords:** Geology, Statistics, Scanning electron microscopy

## Abstract

The key to most subsurface processes is to determine how structural and topological features at small length scales, i.e., the microstructure, control the effective and macroscopic properties of earth materials. Recent progress in imaging technology has enabled us to visualise and characterise microstructures at different length scales and dimensions. However, one limitation of these technologies is the trade-off between resolution and sample size (or representativeness). A promising approach to this problem is image reconstruction which aims to generate statistically equivalent microstructures but at a larger scale and/or additional dimension. In this work, a stochastic method and three generative adversarial networks (GANs), namely deep convolutional GAN (DCGAN), Wasserstein GAN with gradient penalty (WGAN-GP), and StyleGAN2 with adaptive discriminator augmentation (ADA), are used to reconstruct two-dimensional images of two hydrothermally rocks with varying degrees of complexity. For the first time, we evaluate and compare the performance of these methods using multi-point spatial correlation functions—known as statistical microstructural descriptors (SMDs)—ultimately used as external tools to the loss functions. Our findings suggest that a well-trained GAN can reconstruct higher-order, spatially-correlated patterns of complex earth materials, capturing underlying structural and morphological properties. Comparing our results with a stochastic reconstruction method based on a two-point correlation function, we show the importance of coupling training/assessment of GANs with higher-order SMDs, especially in the case of complex microstructures. More importantly, by quantifying original and reconstructed microstructures via different GANs, we highlight the interpretability of these SMDs and show how they can provide valuable insights into the spatial patterns in the synthetic images, allowing us to detect common artefacts and failure cases in training GANs.

## Introduction

Many geological phenomena within the Earth result from physicochemical processes occurring at length scales ranging from nanometers to micrometres. For example, the motion of tectonic plates is associated with the movement of atomic imperfections (i.e., dislocations) within individual mineral grains (e.g.,^1^). Likewise, the transport of fluids within the crystalline lithosphere^2,3^ and reservoirs for the storage of $\hbox {CO}_\text {2}$^4^ and hydrogen^5^ are governed by processes occurring at grain contacts and within microscopic pore spaces. However, it is not the individual dislocation nor pore that controls phenomena at larger length scales but the interaction of many dislocations and pores in tandem. Hence, to understand and model geological processes at larger length scales, we need to (i) quantitatively characterise microstructures that both include existing samples and generalise beyond them and (ii) establish a link between these microstructures and the physical properties of rocks across length scales.

In the past decades, rapid advances in imaging technologies have made it possible to characterise earth materials at different length scales. For example, electron backscattered diffraction is a 2D imaging technique used to describe polycrystalline rocks and gives valuable information such as the lattice-preferred orientation of minerals in multiphase systems, grain shape, and distribution at different scales^6,7^. Scanning electron microscopy (SEM) utilising backscattered electron (BSE) imaging is another technique that can be used to acquire high-resolution images of large areas of rock surfaces. However, applications of such 2D imaging techniques remain limited as many geological processes such as rock deformation and fluid transport in porous media are inherently volumetric.

X-ray tomography is a widely used technique to obtain three-dimensional images of rock microstructures, which provides detailed information about internal structures with a maximum pixel size of $\pm\, 0.5\,\upmu \hbox {m}$ which would not be sufficient to resolve much smaller features observed in complex heterogeneous media such as carbonates^8^, shales^9^ or dense crystalline rocks. In such circumstances, techniques such as focused ion beam nanotomography (e.g.,^10,11^) can be employed, which acquires images with nanometer pixel size but at the expense of the field of view (FoV). This tradeoff highlights the inherent limitation of imaging technologies; resolution and FoV are in direct competition. Moreover, while tiny structures may control the overall behaviour of a given medium, the modelling domain (i.e., FoV) needs to be sufficiently large to be representative of the whole system^12^.

Another challenge in heterogeneous systems is that the microstructural properties can significantly vary from one sample to another, so their variabilities also need to be evaluated to have a more realistic model. The variability assessment is typically done by conducting several imaging experiments or numerical simulations on different samples, allowing us to obtain a distribution over larger samples and capture the heterogeneity of the medium (e.g.,^13^). However, acquiring large image datasets comes with high costs and a severe time penalty.

Two steps need to be taken to go beyond the limitations of available imaging techniques and bridge the gap across different scales. First, samples of complex earth materials need to be imaged with sufficient resolution (to capture the smallest features of interest in a given system) and quantified using reliable statistical tools- a process known as microstructure characterisation. The second step is to reconstruct synthetic but realistic images of the microstructure at a representative scale which is statistically equivalent to the original sample. Thus, statistical characterisation is a key here to assess how similar the original and synthetic images are in terms of structural and morphological properties. The fundamental underlying assumption of this approach is that the geometrical patterns of a limited number of samples are representative of a large class of materials sharing those patterns. As such, these patterns, often represented in terms of spatial statistics, must be implicitly or explicitly exploited in the reconstruction process. Thus, the reconstruction of heterogeneous media is an inverse problem in which a limited amount of microstructural information is used to (re)construct realistic microstructures and evaluate macroscopic properties^14,15^. Consequently, image reconstruction has become an essential aspect of digital rock physics to produce representative samples for upscaling, multi-scale modelling, and uncertainty assessment.

Several methods have been developed for image reconstruction in recent years, which can be grouped into two main approaches: stochastic methods and deep-learning-based reconstructions. Despite their differences, both methods should be supplemented with microstructure characterisations of the system. Characterisation refers to statistically quantifying and representing the morphology of a system using spatial correlation functions, also known as statistical microstructure descriptors (SMDs)^16^. Traditional stochastic methods take such correlation functions as an input and construct a synthetic microstructure with the same characteristics. On the other hand, although deep-learning methods do not require such information a priori, it is necessary to compute the correlation functions from original and reconstructed images to evaluate the reconstruction accuracy.

Most research on stochastic methods formulates the image reconstruction as an optimisation problem in which *n*-point correlation functions, defined as the probability of *n* random points to lie in a phase of interest (e.g., solid, liquid, or void), are calculated from original images and used as target functions. Next, these methods seek to reconstruct a medium for which the calculated correlation function(s) matches the target function(s) derived from the original image. This match can be obtained by applying stochastic optimisation techniques such as simulated annealing (SA)^14,17,18^. While such a framework is shown to be able to reconstruct single-scale microstructures such as Fontainebleau sandstone using a simple two-point correlation function^17^, it fails in the case of multi-scale complex heterogeneous systems since it only captures the largest scale features^19,20^. Recently, Karsanina et al.^21^ proposed a novel hierarchical optimisation approach to incorporate two-point correlations of different scales in SA for reconstructing coarse and fine microstructures in a single image.

An alternative to two-point correlation is to employ multi-point statistics (MPS), or high-order *n*-point correlation functions ($n\ge 3$). These methods have been used for 3D image reconstruction from 2D images, showing to be more effective in long-range connectivity^22,23^. While reconstructing more realistic images, these methods and improved variants^24–26^ are computationally costly and limited to isotropic media. Chen et al.^27^ developed a set of hierarchical descriptors, termed *n*-point polytope functions, which successively capture higher-order correlations of a given phase in an image. In contrast to MPS, these polytope functions can be computed significantly faster since only the probability of *n* vertices of a randomly- or user-selected regular polytope (e.g., triangle, square, hexagon for *n* = 3, 4, and 6, respectively) is considered, i.e., they can be seen as a subset of *n*-point correlation functions with a fixed edge length. It has been shown that incorporating these higher-order correlation functions in SA as target functions will improve the reconstruction accuracy. However, adding more correlation functions increases the computational costs and makes convergence harder to achieve during SA^28^.

In recent years, the advent of deep learning (DL) has opened up unprecedented opportunities and insight into image reconstruction. Generative adversarial networks (GANs) are among the advanced DL-based generative models which have been successfully applied for 2D^29^ and 3D^13,30–34^ microstructures reconstruction. Notably, a growing body of literature has recently investigated 2D to 3D image reconstructions intending to infer 3D morphological and structural properties using features extracted from 2D images in specific orientations^35–38^.

Despite showing promising results, training GANs stably and efficiently is a non-trivial task. Some of the challenges and different approaches to meet them are provided in the following sections. In this work, we employ three different variants of GANs for microstructure reconstruction: deep convolutional GAN (DCGAN)^39^, Wasserstein GAN with gradient penalty (WGAN-GP)^40,41^, and StyleGAN2 with adaptive discriminator augmentation (StyleGAN2-ADA)^42^. We first compare the performance of a well-trained GAN to stochastic method to reconstruct two-dimensional electron microscopy images taken from two of the most common fluid-rock interactions within the Earth’s lithosphere; (1) the hydrothermal alteration of feldspar in igneous rocks and (2) the hydration of upper mantle rocks to induce serpentinisation. While only two-point correlations are used in SA as a target function, polytope functions are calculated to evaluate the accuracy of both methods in reproducing higher-order structural information in the systems. To our knowledge, previous studies only used two-point correlation and a subset of the so-called Minkowski functionals (e.g., specific surface area and Euler connectivity) to assess image reconstruction performance - none of these choices, however, have explicitly addressed how machine-learning-based reconstructions perform in reproducing high-order spatial correlations. When determining how well we can reconstruct higher-order complexity, our results show that while a reconstructed microstructure can have the same two-point correlation as the original one, they can be morphologically different. Therefore, it is necessary to couple higher-order correlation functions with reconstruction methods. Furthermore, we compare the performance of three types of GANs on representative images using the proposed polytope functions. First, we assess quantitatively the accuracy of GANs by computing the error between SMDs of original and reconstructed microstructures. Then, we interpret the SMDs derived from each GAN and argue how such an analysis can shed light on different geometrical patterns, as well as artefacts reproduced by different GANs.

## Theoretical approach

### Rock samples and dataset

We focus on two commonly occurring rock types affected by fluid-rock interaction. The first example is an altered igneous rock representative of fluid-rock interactions of the Earth’s crust^2^ and the second example is a partially serpentinised peridotite representative of alteration within the Earth’s uppermost mantle^43^. For simplicity, we refer to these two rock samples as meta-igneous rock and serpentinite from here on.

For the meta-igneous rock, a small core was drilled with a diameter and height of 2.5 mm and 1 cm, respectively. The core was cut, and the surface was imaged in backscattered electron (BSE) mode using the Zeiss Atlas software installed on a Zeiss Gemini 450 SEM. Zeiss Atlas allows large-area BSE imaging of up to several centimetres. Acquisition conditions were 20 kV acceleration voltage and 2 nA beam current. The pixel size was set to 50 nm. Subsequently, a region of interest with dimensions of 17,920 by 54,784 pixels (0.9 mm by 2.7 mm) was imaged.

For the partially serpentinised peridotite, the Atlas software-based BSE imaging approach was utilised on various samples from selected Norwegian peridotites previously described in^43^ and^44^. The rock samples are characterised by a lizardite-mesh texture with remaining olivine and secondary magnetite. The serpentinisation process produces a fracture network creating pathways for fluid flow. In this case, a small part of a thin section was scanned with an acceleration voltage of 15 kV, 2 nA current, and pixel size of 500 nm, resulting in a large image of dimensions 15,000 by 30,000 pixels (7.5 mm by 15 mm).

To remove the noise and artefacts that are often present in raw images, the acquired grayscale BSE images were first denoised by applying an edge-preserving denoising algorithm known as bilateral filtering. This filter smooths an image by averaging pixels based on their spatial distance and pixel value similarities. For more details, please see the original work by^45^ and the scikit-image Python package’s documentation^46^. The filtered images were subsequently segmented into binary images where a pixel value of 1 corresponds to the phase of interest, which is the reaction-induced pore network in igneous and the fracture network in serpentinite samples, respectively. Image segmentation employed a convolutional neural network (CNN) with U-Net architecture^47^. This method is supervised deep learning and thus, requires previously annotated images for training. These labeled images were created using the ilastik software; an interactive software developed for image classification and segmentation^48^. It is worth mentioning that although one can do the image segmentation using ilastik alone, the benefit of training a CNN is that, once trained, it can be used to quickly segment future images either directly (if the sample and imaging conditions are the same) or using transfer-learning.

### *n*-point correlation functions

Correlation functions have been proposed as an effective means to describe complex heterogeneous microstructures mathematically. The most widely used microstructural correlation function is the two-point correlation, $S_2 (r)$, which is the probability *P* of two random points of distance *r* to occur in the same region of phase *i*, $V_i$, within a *d*-dimensional space $R^d$^49^:

1 $$\begin{aligned} \qquad S_2^{(i)}(r) = P(x\in V_i \ , x+1 \in V_i )\ \text {for}\ x\ and \ V_i \in R^d \qquad \end{aligned}$$

where *x* is an index showing the location of a pixel in the microstructure image. This is a radial form of two-point correlation calculated by averaging the functions in horizontal and vertical orientations for a statistically isotropic and homogeneous system. According to this definition, the probability of one random point (*r* = 0) to occur in phase region $V_i$ is a one-point correlation corresponding to the volume fraction of that phase, i.e., $S_1^{(V_i)} = S_2^{(V_i)} = \phi _i$.

**Figure 1:**
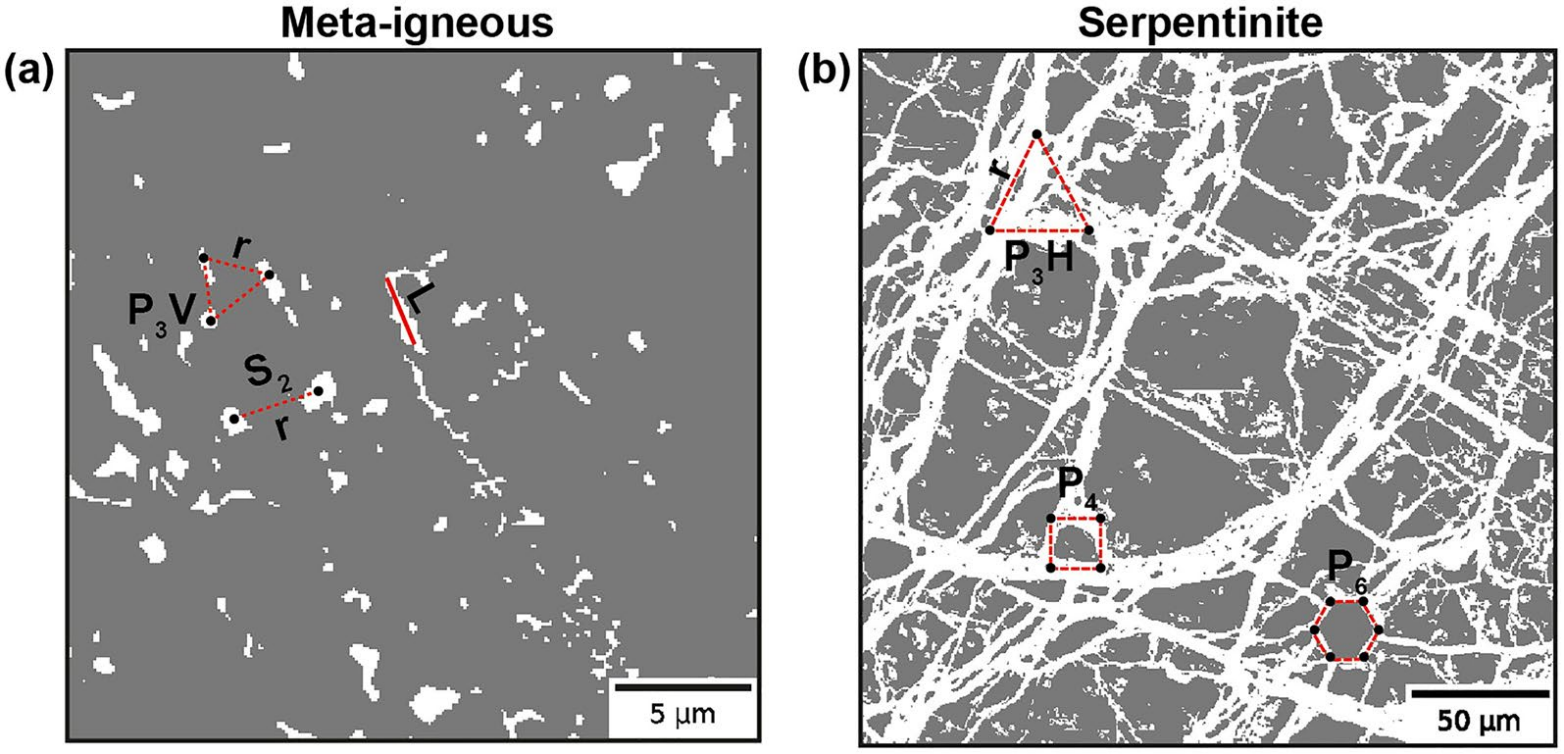
Illustration of polytope functions in meta-igneous rock (**a**) and serpentinite (**b**) samples. $P_{3H}$, and $P_{3V}$ indicate the probability of vertices of horizontal and vertical triangles, and $P_4$ and $P_6$ are those of a square and hexagon, respectively. L is the lineal-path function.

As mentioned earlier, two-point statistics is not sufficient to uniquely characterise complex systems containing higher-order spatial correlations in different locations of an image. In such cases, an *n*-point correlation function $S_n$ can be defined as a probability of *n* random points to lie in a given phase. While a complete description of the medium can be achieved by a set of $S_n$ with $n=1,2,3,\ldots, \infty$, calculating and storing probabilities of all possible *n*-points statistics is computationally intractable. Thus, to sample high correlations from digitally-sampled microstructures, a compromise must be made between geometric completeness and algorithmic practicality. One such approach to capture complex microstructures uses *n*-point polytope functions, defined as a probability of *n* vertices of a random regular *n*-point polytope having a given edge length that occurs within the same phase (Fig. 1).

The polytope functions capture partial higher-order *n*-point correlations of a specific phase and can be directly computed from 2D or 3D images and incorporated in a stochastic optimisation method for image reconstruction^27^. As shown in Fig. 1, for $n=2$, the two-point polytope function is the same as $S_2$. However, for $n=>3$, the higher-order polytope functions can be seen as a subset of the *n*-point correlation functions, which can be efficiently computed as the edge length (*r*) is the only variable. Also, to further quantify morphological patterns, we use a lineal-path function *L*^50^. This function is a statistical morphological descriptor defined as the probability that an entire line of the length *r* occurs in the same phase. Thus, it can provide additional insight into microstructure connectivity and linear clustering. A scaled version of these functions, known as scaled autocovariance function, has been introduced by^49^ and is related to the $P_n$ functions via:

2 $$\begin{aligned} F_n(r) = \frac{P_n(r)-\phi ^n}{\phi - \phi ^n} \end{aligned}$$

where $\phi$ is the phase fraction. According to this equation, $F_n (r=0) = 1$ and $F_n (r \rightarrow \infty ) = 0$. The latter is obtained as for $r\rightarrow \infty$, we have $P_n \approx \phi ^n$. Although both $F_n$ and $P_n$ functions show the same behaviour for a given microstructure, it is more convenient to use the scaled autocovariance as it is normalised by the phase fraction - meaning that they describe geometric patterns independently from a given phase’s volume fraction.

### Stochastic reconstruction

In this study, we use the stochastic Yeong-Torquato-based reconstruction algorithm developed by Jiao and co-workers as a benchmark^14,17^. This approach formulates image reconstruction as an optimisation problem in which reconstruction is performed by minimising a cost function using simulated annealing (SA) optimisation^51^. The algorithm starts with a two-point correlation $S_2$ of an original image as a target function and a random initial system configuration with the same volume fraction. An energy function is defined as the sum of the square errors between the target function and correlation function of the proposed configuration $\hat{S}_2$:

3 $$\begin{aligned} E= \sum _{r}{\left[ \hat{S}_2 - S_2(r)\right] } ^2 \end{aligned}$$

To evolve the random reconstruction towards the original image, the values of two random pixels associated with different phases (i.e., black and white pixels) are exchanged, ensuring that the volume fraction of both phases is preserved. Hence, a new energy $E_{\text {new}}$ corresponding to the new configuration, and the energy difference between two successive configurations $\Delta E= E_{\text {new}}- E_{\text {old}}$ are calculated. Finally, the pixel exchange is accepted according to the Metropolis acceptance rule:

4 $$\begin{aligned} p(\Delta E) = \left\{ \begin{array}{ll} 1,&{} {\text {if}}\;\hbox {E}_{\text {new}} < \hbox {E}_{\text {old}}\\ exp(-\Delta E/ T), &{} \text {if } \hbox {E}_{\text {new}} \geqslant \hbox {E}_\text {old} \end{array}\right. \end{aligned}$$

where $p(\Delta E)$ is the acceptance probability of the pixel exchange, *T* is an imaginary temperature that is initially set to a high value and decreases by a factor of $\alpha$ (selected to be less than but close to 1) after each annealing stage of the algorithm i.e., $T= \alpha \times T_0$. Accordingly, when the temperature is high at the initial steps, the acceptance probability of the pixel exchange can be higher even if $E_{\text {new}} \geqslant E_{\text {old}}$, i.e., the error between target and sampled two-point correlation in the new configuration is higher than the old one. Thus, the probability of accepting a bad configuration is higher. This helps to explore the whole solution space and prevents the algorithm from trapping in local minima. These steps are repeated until the energy (error) of the reconstructed image is less than a predefined threshold value or a maximum number of iterations is reached. In this study, $1e^{-7}$, 0.97, and 250 were selected for the initial temperature ($T_0$), the decreasing factor ($\alpha$), and the number of iterations, respectively.

Although the Yeong-Torquato SA is a well-known, flexible optimisation method that allows the incorporation of correlation functions to improve the reconstruction process, the computational costs significantly increase by including additional functions or dimensions - because they must be sampled at any iteration. And, similar to most stochastic optimisation methods, inference (reconstructions) accuracy is achieved after relatively large numbers of iterations. Hence, we only use the two-point correlation function as a benchmark against our GAN models.

### Generative adversarial networks(GANs)

GANs are unsupervised generative algorithms based on game theory^52^, which can directly learn complex high-dimensional probability distributions from the input data. The term *adversarial* originates from the fact that GANs are composed of two neural networks competing against each other: a generator (G) and a discriminator (D). The generator’s task is to generate realistic images from the data distribution $p_{data}$. This is done by transforming noise (i.e, random) vectors *z* into $x=G_{\theta } (z)$, with $\theta$ being a set of learnable parameters. These noise vectors, also known as latent space, are random variables usually sampled from a normal distribution. During training, the discriminator $D_\theta$, which is a binary classifier, receives both real images (from $p_{data} (x)$) and reconstructed images (from $p_{model} (x)$), and then learns to maximise the probability of correctly labeling reconstructed (with label= 0) and real images (with label= 1). At the same time, the generator is trained in such a way that a chosen discriminator metric e.g., $log(1-D(G(z)))$ is minimised (i.e., to ’fool’ the discriminator into classifying the reconstructed image as real with $D(G(z))= 1$. Mathematically, the cost function for such a GAN, which is called DCGAN when convolutional layers are used in G and D, is a minimax game with value function *V*(*G*, *D*)^52^:

5 $$\begin{aligned} \underbrace{min}_G \,\underbrace{max}_D V(D,G) = \mathbb {E}_{x\sim p(data)}[(logD(x))] + \mathbb {E}_{Z\sim p(Z)}[log(1-D(G(Z)))] \end{aligned}$$

#### Challenges in training GANs

Although GANs have been successfully applied to reconstruct a wide range of images, training GANs stably and efficiently is non-trivial. The training involves achieving a Nash equilibrium to a non-cooperative game between the generator and the discriminator, each of them having its cost function: $J^{(D)} (\theta ^{(D)},\theta ^{(G)})$ and $J^{(G)} (\theta ^{(D)},\theta ^{(G)})$ for the discriminator D and the generator G, respectively. A Nash equilibrium is reached when a combination of parameters $(\theta ^{(D)},\theta ^{(G)})$ is found so that $J^{(D)}$ is minimum with respect to $\theta ^{(D)}$, and $J^{(G)}$ is minimum with respect to $\theta ^{(G)}$. Finding these parameters to reach Nash equilibrium is difficult because a change in $\theta ^{(D)}$ to reduce $J^{(D)}$ may increase $J^{(G)}$, and similarly, a modification to $\theta ^{(G)}$ for minimising $J^{(G)}$ can increase $J^{(D)}$. Therefore, although the two players might reach an equilibrium in some cases, updating the parameters of both models does not necessarily lead to stable and convergent training. However, this is not a specific issue of GANs, but it is a general problem with game-theory-based approaches.

Mode collapse is another common issue in GANs, which occurs when the generator collapses to a set of parameters $\theta$ that leads to reconstructing the same images, i.e., mapping different noise vectors into the same output^53^. The reason is that the discriminator receives and analyses each image independently. Therefore, when it cannot differentiate between real and reconstruction for a specific example, the generator updates its parameters to create more of that example and *win* the game.

Several heuristic methods are proposed to overcome these challenges and improve training stability. Some effective techniques are: feature matching, minibatch discrimination, historical averaging, one-sided label smoothing, and visual batch normalisations^54^. Adding Gaussian noise to the discriminator’s input and label switching can also stabilise the training process^30^, though the greater statistical conditions and implications of this approach are still to be researched. Furthermore, some studies have investigated the use of other distance metrics and value functions than binary cross-entropy (BCE) (Eq. 5). Arjovsky et al.^40^ used Earth-mover or Wasserstein-1 distance to measure the distance between the probability functions of real and reconstructed images. This method, known as WGAN, can prevent mode collapse and improve stability and convergence behaviour by forcing the gradient of the discriminator in a constrained space. This can be done by applying a weighted clipping or a gradient penalty, with the latter method known as WGAN-GP^41^. In this paper, we use WGAN-GP to enforce the output of the discriminator in [− 1, 1]:

6 $$\begin{aligned} {\underbrace{min}_G \,\underbrace{max}_D V(D,G) = \mathbb {E}_{x\sim p(data)}[(D(x))] - \mathbb {E}_{Z\sim p(Z)}[D(G(Z)))]+ \lambda \mathbb {E}_{\hat{x}}[{({\big \Vert \nabla _{\hat{x}}D(\hat{x}) \big \Vert }_2 -1)}^2]} \end{aligned}$$

where $\hat{x}$ is a mixture of real and the reconstructed image from the generator network calculated via $\hat{x}= \varepsilon (x)+(1-\varepsilon ) G(z)$, in which $\varepsilon$ is a random number from a uniform distribution *U*[0, 1]. $\lambda$ is the coefficient of the gradient penalty and here is set to 10. The rationale for choosing this value can be found in the Supplementary information.

Miyato et al. 2018^55^ used spectral normalisation in GAN (SNGAN) to solve the stability problem. This normalisation technique was later coupled with WGAN by^33^ for 3D image reconstruction of electrodes. More recently, Karras et al. proposed the style-based GANs, namely StyleGAN^56^, StyleGAN2^57^, StyleGAN2-ADA^42^, and StyleGAN3^58^. In all these versions, the style refers to the variations in images representing different levels of detail (e.g., global and local features). Experiments and analyses on different datasets suggest that these models can generate high fidelity and more diverse images of larger size, with more control over styles. Despite the differences, all StyleGANs consist of a mapping network and a synthesis network. The role of the mapping network is to map the random noise vector *z* into intermediate vectors *w*, which allows the generator to learn disentangled, less correlated features. However, the synthesis network is different in these variants. The StyleGAN uses progressive growth architecture^59^ with adaptive instance normalization (AdaIn) applied after each convolution layer. However, the AdaIN is found to be a source of artefacts and is modified in StyleGAN2. Also, instead of progressive growth, StleGAN2 employs skip connection and residual networks in generator and discriminator, respectively. Here, we implement the StyleGAN2-ADA which is similar to StyleGAN2 in terms of the loss function and network architecture, but with an additional augmentation pipeline that helps to improve stability and avoid the overfitting problem, especially when the number of training images is limited.

#### Training workflow

**Figure 2:**
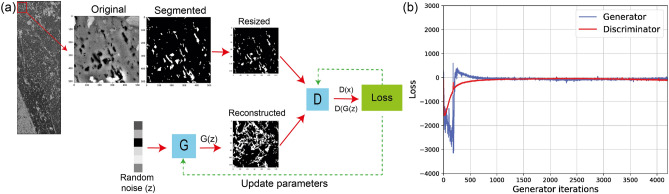
Training WGAN-GP with BSE images of the meta-igneous rock. (**a**) Schematic description of the general workflow. G and D are the generator and the discriminator with architectures described in Table S1. (section) Training dynamics of WGAN-GP show how two losses are converging around zero with generator iterations. In practice, the parameters of the discriminator were updated five times for each generator’s update. The same workflow was used for the reconstruction of the serpentinite.

While different architectures can be employed in GANs, several studies suggest that using convolutional networks in generator and discriminator can improve the fidelity of synthetic images and training performance. Such a network, known as deep-convolutional GAN (DCGAN), was first introduced by^39^. The general workflow used in this study for training different GANs is shown in Fig. 2a. We extracted smaller images from the two samples to prepare sufficient training images. In the meta-igneous sample, a total number of 14,697 images were created by sliding a window of the size 512 $pixel^2$ with a stride of 256 pixels over the original large BSE image. For the serpentinite sample, sliding window size and stride were 1024 pixels and 200, respectively, providing 10,150 training images. These images were then segmented and resized to $128^2$. The smaller window size is selected for the meta-igneous sample because we observed a significant loss of information while downsampling larger images to $128^2$ which was our target size. This target size is chosen for the purpose of comparison of two reconstruction methods since it was the largest size that the stochastic algorithm converged. In the following sections, we show that this image size is not representative, and only GANs can generate synthetic images of representative size. More details about the architecture and hyperparameters used for training GANs used in this study can be found in supplementary information.

Figure 2b displays the evolution of loss functions versus generator iterations WGAN-GP. It can be seen that after 250 iterations, generator and discriminator losses start converging stably. However, since a decrease in generator loss cannot be related to reconstruction quality, an average $S_2$ curve was calculated for real and simulated batch images (batch size = 128) at the end of each iteration. Subsequently, the MSE between two average curves was used as the reconstruction quality criterion. The best results were obtained at iteration 4300 with MSE= $1.66e^{-8}$. The training was performed on two NVIDIA Quadro P6000 GPUs, and the curves converged after 3 h.

## Results

In this section, we first compare the capability of stochastic and a well-trained GAN on BSE images of size $128^2$ obtained from both samples using the workflow shown in Fig. 2a. Then, three variants of GANs are trained on images of size $512^2$ which is shown to be the representative size for both samples. The reconstruction quality is then evaluated in terms of the error between SMDs computed in the original and reconstructed images. In all cases, the average functions of 128 images are calculated and compared.

### Microstructure reconstruction: GAN versus SA

**Figure 3:**
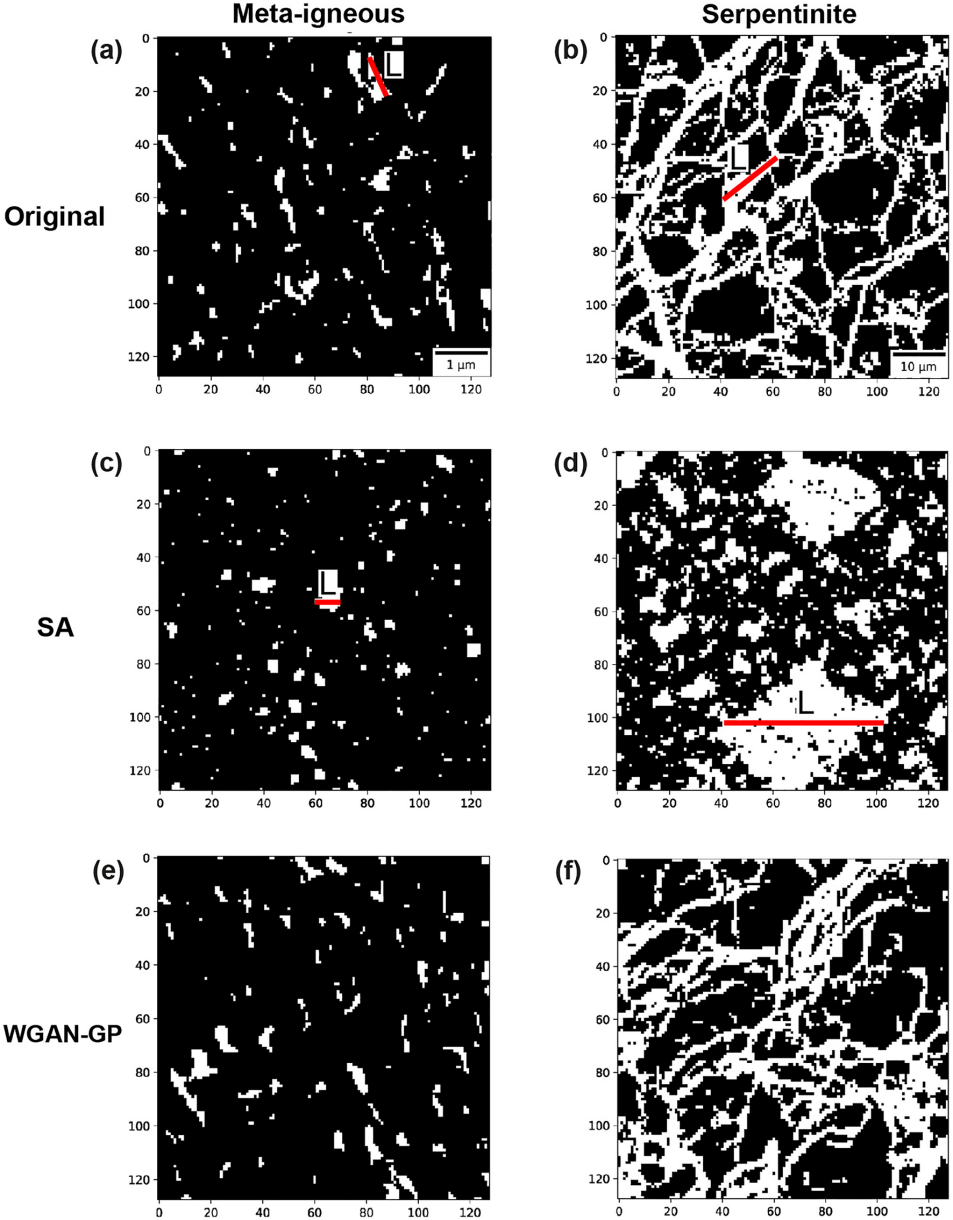
Visual comparison of real (**a**,**b**) with reconstructed images using SA (**c**,**d**) and WGAN-GP (**e**,**f**). Red solid lines illustrate how the lineal-path function *L* can describe the linear clusters and connectivity in the microstructures.

Figure 3 depicts the image realisations obtained from SA and the WGAN-GP and highlights the importance of capturing higher-order correlation functions for generating realistic images. The reason for selecting WGAN-GP is that its training was stable (compared to DCGAN) and relatively fast (compared to the StyleGAN2-ADA). Visual inspection of the reconstructions shows that this GAN can generate more realistic images with similar geometrical and structural features such as shape, size, and orientation. This is consistent with quantitative analysis of polytope functions, showing that different levels of morphological symmetries are reproduced in GAN-reconstructed images.

**Figure 4:**
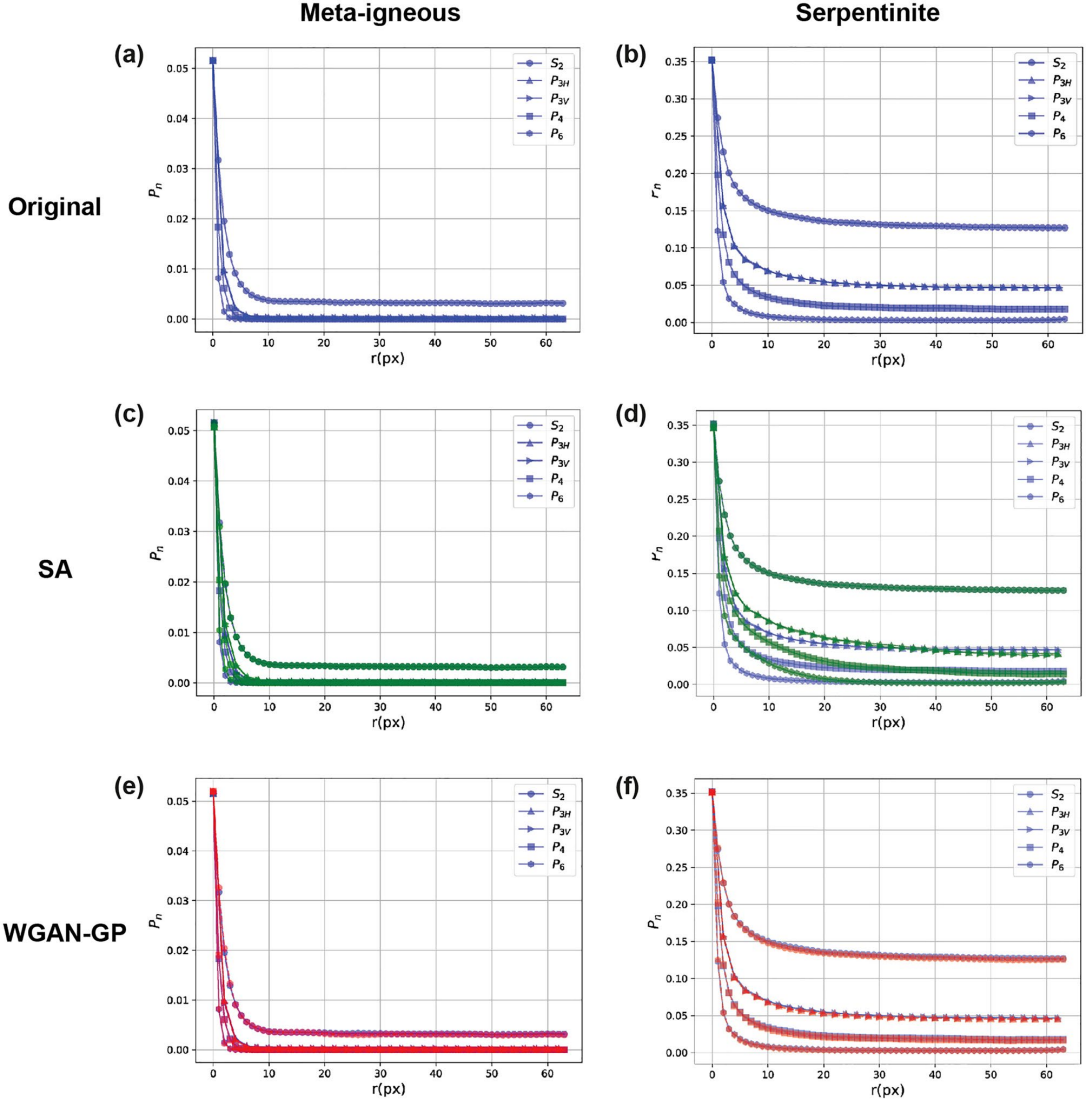
Evaluation of reconstruction performance using SMDs. Quantification of original (**a**,**b**) and reconstructed microstructures using SA (**c**,**d**) and WGAN-GP (**e**,**f**).

Figure 4 presents the $P_n$ polytope functions corresponding to different geometrical correlations. These functions provide new statistical information about the microstructures. Each $P_n$ function provides new statistical information about the microstructures compared to $P_{n-1}$. For example, the $P_4$ function shows the square correlations (patterns) in the sample and adds unique higher-order information to triangular correlations ($P_{3H}$ and $P_{3V}$), which in turn, are complementary to $P_2$. Figure 4a-b show the average $P_n$ functions of the original microstructures in the two samples. In each case, all functions start with the same probability at $r=0$, corresponding to porosity (or area fractions of phases of interest), which are 0.052 and 0.352 for the meta-igneous rock and serpentinite samples, respectively. It can be seen that the correlation functions initially decrease as *r* increases. However, the reduction rate of each correlation function is faster than its lower-order function. This is because, for example, it is less probable that all vertices of a hexagon lie in the same phase compared to those of a triangle with the same edge length. Furthermore, the *r* value in which the curves stabilise shows the average size of the features of interest in the samples i.e., average pore size and fracture width, respectively. Thus, one can infer that the average pore size is < 10 pixels ($\sim\, 0.5$ μm) in meta-igneous microstructures (Fig. 4a), and similarly, the average serpentinite fracture width is 20 pixels ($\sim\,10$ μm) as shown in Fig. 4b.

To compare and evaluate the accuracy of microstructure reconstruction, polytope functions are computed on 128 reconstructed images by the SA and WGAN-GP. Figure 4c,d shows the quantification of microstructures reconstructed via the SA method (green curves). As can be seen, while there is a good agreement between $S_2$ curves of the original and reconstructed images, apparent discrepancies are observed between higher-order correlation functions (i.e., for $n>2$). A small error between $S_2$ functions was expected as this function is used as the target function in the SA algorithm. Figure 4e,f compares the polytope functions calculated from the reconstructed images via WGAN-GP with the original images. The close agreements between all polytope functions indicate that reconstructed images with WGAN-GP contain the higher-order structural information present in the original microstructures. To quantify and compare the reconstruction accuracy of the two methods, the MSE between $F_n$ functions derived from original and reconstructed images are calculated and presented in Table S3. The table highlights that the two reconstruction methods are comparable in capturing $S_2$. However, with respect to higher-order correlations, the errors computed from WGAN-GP are between two (for meta-igneous rock) to three (for serpentinite) orders of magnitude less than the stochastic method.

### Representative elementary size

The main question that needs to be addressed in microstructure reconstruction is determining representative image size capturing structural elements of the system under consideration. It has been shown that the models trained on small images will create pores with artefacts and unrealistic shapes. Although, the larger the size of the training images, the more computationally demanding and less stable the training.

Mosser et al.^13^ proposed to use average grain size and chord length as the minimum training image size. However, a representative elementary size (RES) analysis should be carried out for heterogeneous and complex samples to find an adequate training image size^38^. RES analysis is a methodology to determine the smallest size of a system that is large enough to capture the system’s heterogeneity as a whole^60^. RES analysis is conventionally performed for a particular rock property such as porosity or permeability and is used in upscaling to evaluate the effective macro-scale properties of rocks from a smaller yet representative sample size. Thus, the RES determined by this method can significantly vary depending on the property of interest. Furthermore, this approach involves plotting sample size versus its corresponding calculated property. A common observation is that the property fluctuates widely at small sizes, but it becomes insensitive to size at some point which can be considered the representative size, i.e., the transition between micro- and macro-scale^61^.

**Figure 5:**
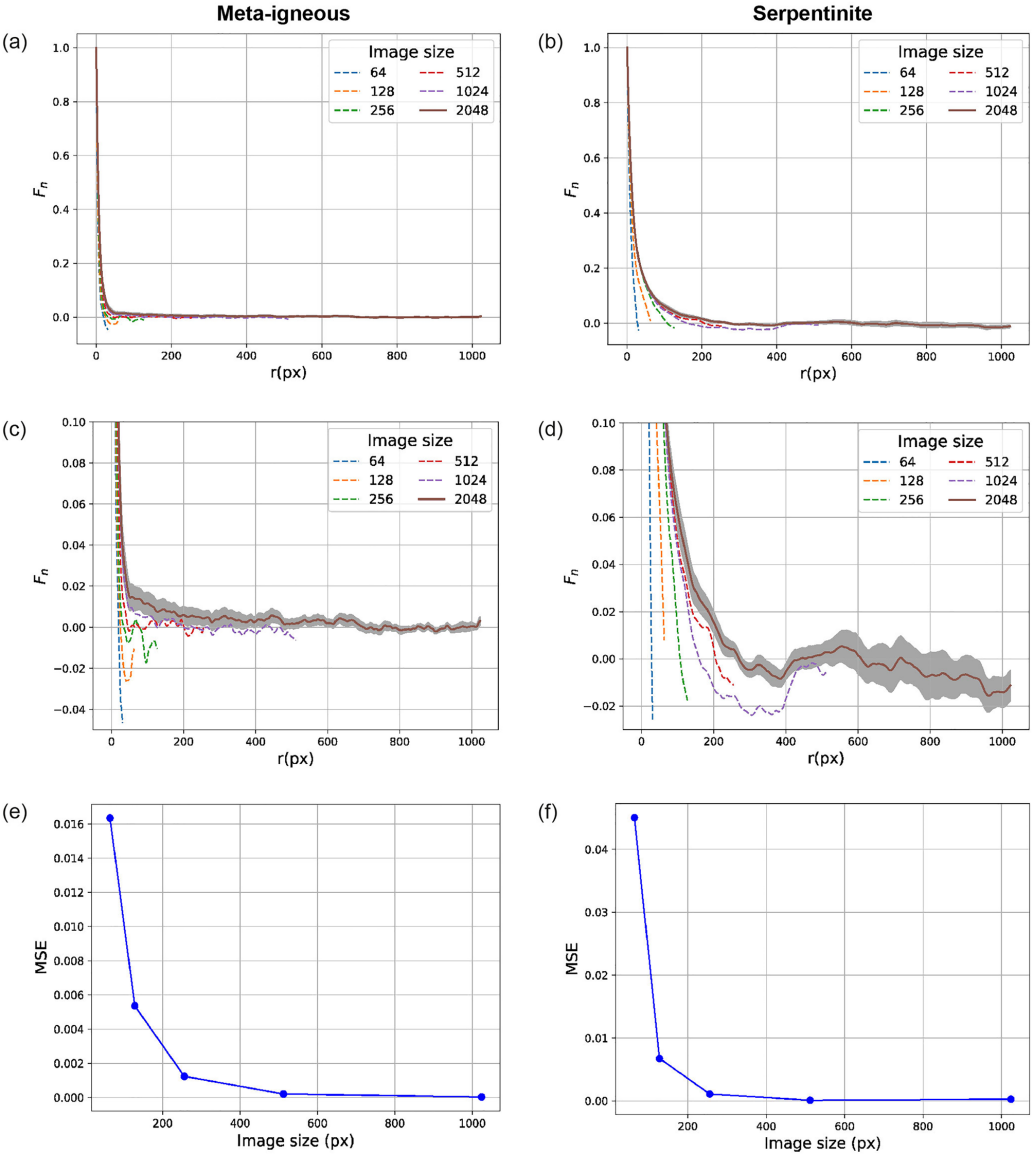
RES analysis by scaled autocovariance function $F_n$ (Eq. 2) calculated for images of different sizes. (**a**,**b**) Are $F_n$ functions computed for the meta-igneous rock and serpentinite samples, respectively, where (**c**,**d**) show the magnified views of (**a**,**d**). Grey shadow indicates the 95 percent confidence levels around the average values of the largest image ($2048^2$ pixels). (**e**,**f**) MSEs are calculated between the average $F_n$ of the largest image and smaller ones.

Here, we rely instead on the two-point correlation function to determine the representative image size - this allows for a material-dependent representative image size. Such a representative size is characteristic of porosity (included as volume fraction in $S_2$), but it is also structurally and topologically representative, which is important for many post-reconstruction analyses such as fluid flow simulations. Our approach consists of computing the average scaled two-point correlation ($F_2$) for 30 images of different sizes randomly selected from our original large BSE images of both samples (Fig. 5a,b). Then, MSEs between the largest image (i.e., of size $2048^2$ pixels) and smaller images at the overlapping range are calculated (Fig. 5e,f). It can be seen that the $F_2$ curves for the images smaller than 512 pixels show entirely different patterns (Fig. 5c,d), leading to more errors while the MSE does not decrease significantly beyond 512 pixels. Thus, an image of size $512^2$ pixels can be considered representative for both samples.

### Reconstruction of representative microstructures

In this section, we present the results of representative image reconstruction using different GANs. The DCGAN is selected as a benchmark and its performance is compared with WGAN-GP and StyleGAN2-ADA. However, SA algorithms did not converge with such a large system because the microstructural degeneracy of the system exponentially increases with the number of pixels. This leads to a rough and complex energy landscape (i.e., model posterior or solution space) associated with SA optimisation, thereby exploring the solution space to find the minimum global energy becomes numerically challenging^19,62^.

**Figure 6:**
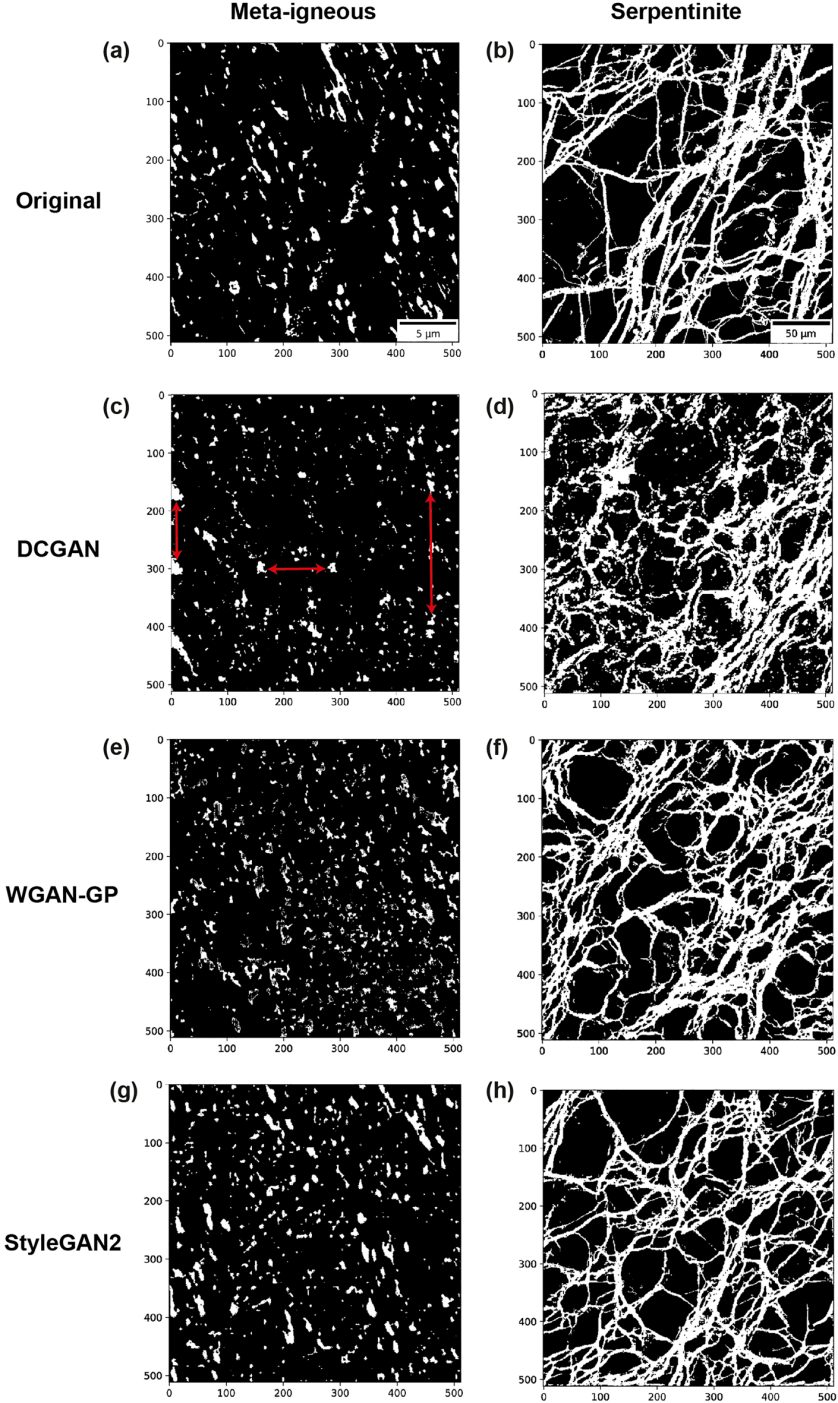
Examples of image reconstructions of meta-igneous (first column) and serpentinite (second column) with representative size using GAN models. (**a**,**b**) Original microstructure, (**c**,**d**) DCGAN-reconstructions, (**e**,**f**) WGAN-GP- reconstructions, (**g**,**h**): StyleGAN2-ADA. The red arrows in (**c**) indicate the pores repeated at specific distances ($r\approx 128$ and $r\approx 256$).

The first column in Fig. 6 shows original and reconstructed microstructures of the pore network in meta-igneous rock. The results indicate that DCGAN can generate realistic microstructures in terms of pore size and pore orientation of the system. However, on closer inspection, it can be seen that some patterns (red arrows in 6c) are repeated. These repeated patterns are not observed in reconstructed images by WGAN-GP (Fig. 6e) and StyleGAN2-ADA (Fig. 6g). In addition to this visual inspection, the quantification of reconstructed images using SMDs confirms the superior performance of these two variants to DCGAN (see discussion).

The results of reconstructions for the complex fracture network in the serpentinite sample can be compared in the second column of Fig. 6. From the images, we can see that DCGAN is able to capture the main structures, but the connectivity of fractures is better reproduced by WGAN-GP (Fig. 6f) and StyleGAN2-ADA (Fig. 6h). Moreover, the trained DCGAN was not able to generate diverse images i.e., all 128 reconstructed microstructures were very similar. As mentioned before, this is a common challenge in training GANs known as the mode collapse. Figure S1 illustrates how quantifying reconstructions using two-point correlation ($S_2$) allows us to evaluate the diversity of synthetic images, which can be also monitored during training to detect this phenomenon.

## Discussion

One of the main aims of this paper was to illustrate the necessity of using higher-order correlation functions, which are common in material science to characterise microstructures, in training and reconstruction quality evaluation. We argue that these functions, as an external tool to the loss function, can encode more structural and topological information than the metrics used in previous studies (e.g., porosity, pore area, Euler connectivity, etc.), with the additional advantage of being interpretable. In this section, we show how these SMDs can assist us in understanding microstructure and immediately detect spatial patterns and/or artefacts in the reconstructions.

Our results show that capturing these geometrical correlations is an inherent capability of well-trained GANs. The term *inherent* here refers to the fact that the GAN has not been trained to fit higher-order polytope functions. The information used by GAN during the training were (1) a great number of training images, and (2) the MSE error between $S_2$ of the original and reconstructed images as a stopping criterion. The above-mentioned capability of GANs may be explained by the fact that there are multiple layers of convolutions in the discriminator, and each of them encodes spatial information from the images in the form of feature maps. In order for this nonlinear set of parameters to represent our sample geometry, the training data and chosen loss functions are the user-supplied priors - which the GAN relies upon during training to achieve network weights that lead to accurately-inferred reconstructions. In that process, the generator learns to simulate images containing those spatial correlations to *fool* the discriminator - informed by the large prior number of training images extracted from our imaged samples.

### Reconstruction performance: GAN versus SA

Visual inspection of the reconstructions provided in Fig. 3 shows that WGAN-GP can generate more realistic images with similar geometrical and structural features such as shape, size, and orientation in both samples. This is consistent with quantitative analysis of polytope functions (Fig. 4), showing that different levels of morphological symmetries are reproduced by WGAN-GP.

**Figure 7:**
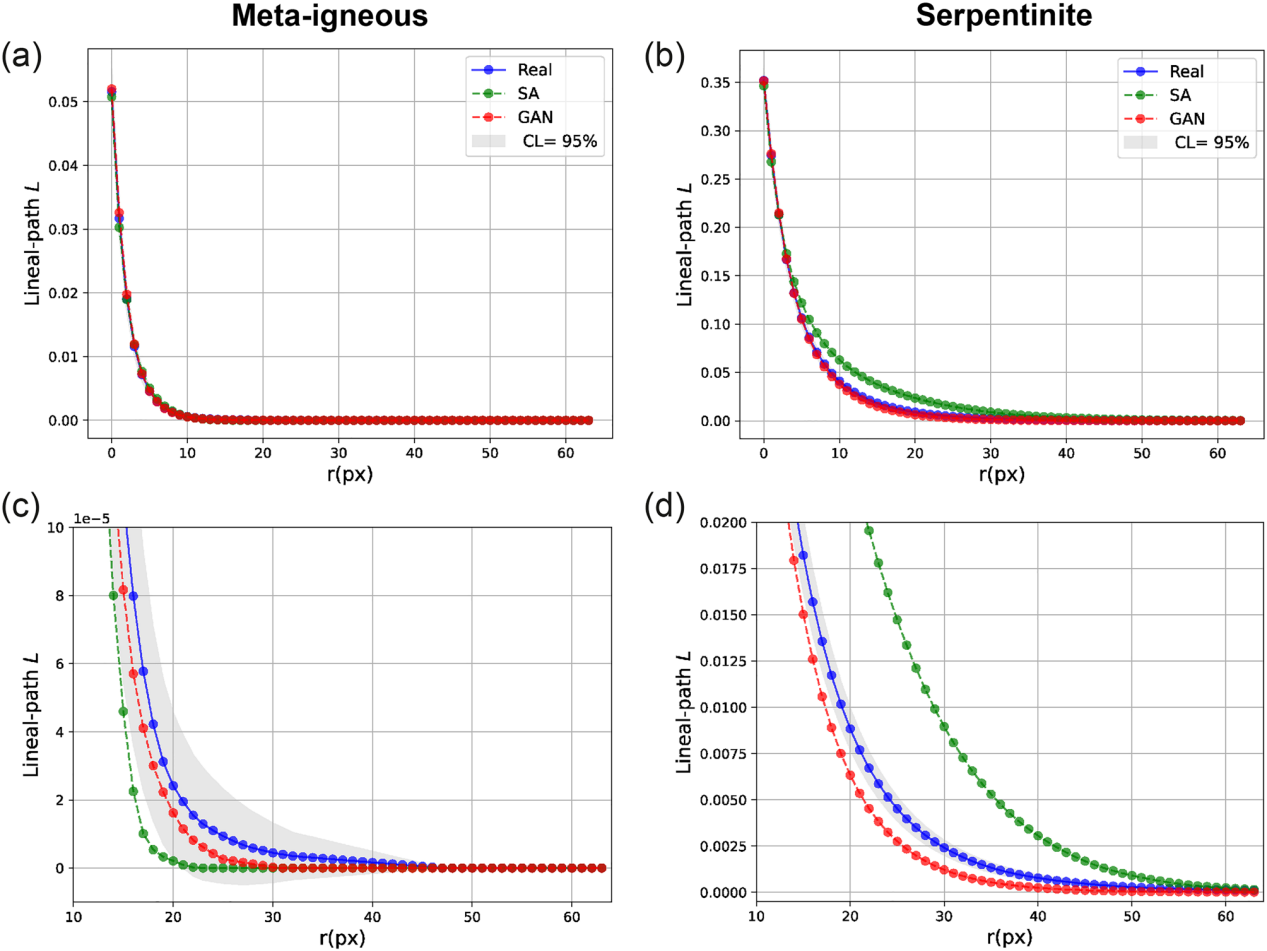
The average lineal-path functions *L* calculated from real (blue), SA-reconstructed (green), and GAN-reconstructed (red) images. Grey areas in (**c**,**d**) are 95 percent confidence bounds around real curves. A better match between curves is derived via GAN reconstructions in both samples. CL = confidence level.

**Figure 8:**
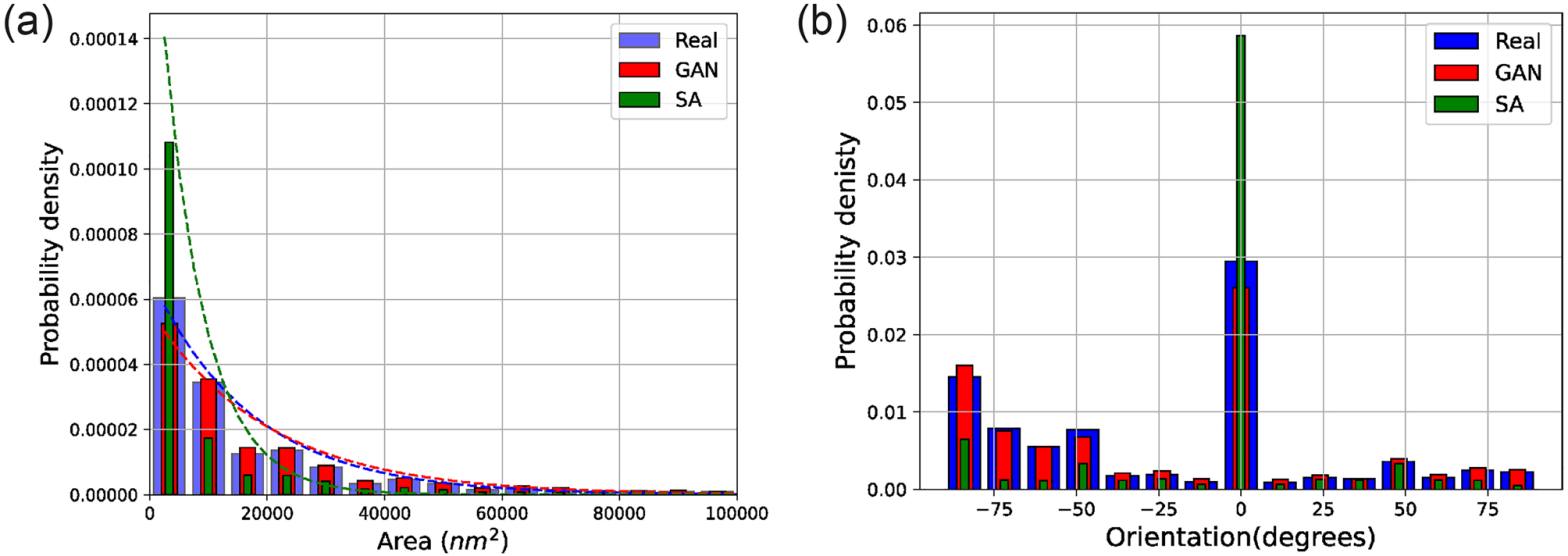
Comparison of real and reconstructed images in terms of pore properties in the meta-igneous rock. (**a**) Probability density of the pore area was calculated from 128 randomly sampled images. Dashed lines show the estimated probability density functions fitting the data. (**b**) Distribution of pore orientations between − 90 (clockwise) and + 90.

In the case of the meta-igneous rock, we observe that the pores reconstructed by SA (Fig. 3c) are circular and smaller than the original pores, lacking the preferred orientation apparent in the original microstructure. This observation is also confirmed by determining the average lineal path *L* computed from 128 images, as presented in Fig. 7. Although there is a close agreement between the *L* of original and reconstructed images (Fig. 7a), zooming into the flat part of the curves (Fig. 7c) reveals that SA has underestimated the *L* (i.e., the linear connectivity of pores) in the system. In particular, instead of the elongated pores in the original images, round-shaped isolated pores of smaller diameters are reconstructed. Figure 8 compares the probability distributions of the area and orientation of the major axis of pores in the real and reconstructed images of the meta-igneous rock sample, computed by the label analysis tool within the Thermo Fisher Scientific AVIZO software. From Fig. 8, we can conclude that WGAN-GP reproduces pores of similar size and orientation to the natural system, whereas there is a significant difference between SA-reconstructions and the natural microstructure.

Moreover, it is also apparent from Fig. 3 that the SA-reconstructed images are entirely different from the original ones in serpentinite fracture networks. Specifically, circular pores of large diameters are reconstructed instead of reproducing a network of fractures with varying widths and orientations (Fig. 3d). This observation is in agreement with our earlier results, which show that SA overestimates all higher-order polytope functions (Fig. 4b) and lineal-paths (Fig. 7d) with errors up to three-order of magnitudes greater than WGAN-GP, leading to reconstructed images that have no resemblance to the original microstructure.

Unrealistic reconstructions obtained by the SA are due to the microstructural degeneracy of the $S_2$ function, i.e., a number of different microstructures can be compatible with the given target function $S_2$ (Eq. 3), resulting in a near-zero energy^62^. This means that the $S_2$ does not contain sufficient information to characterise the system uniquely - particularly with regards to higher-order structures. However, it can be seen that SA performs much better in the meta-igneous rock sample than in the serpentinite sample. This may be explained because the serpentinite microstructure is of higher geometrical complexity, i.e., it contains higher-order correlations at different length scales (Fig. 4b). In contrast, there are not many higher-order correlations in the porous meta-igneous rock system at longer ranges (Fig. 4a), in which polytope correlations become zero at short ranges.

In addition to higher accuracy, the computational reconstruction time of GANs is much less than for the SA method. This time difference is expected given the nature of stochastic optimisation versus the machine-learning approach. While stochastic optimisation yields a single reconstruction by numerically sampling a model posterior distribution iteratively (conducted independently for each reconstruction), GAN directly yields samples from the model posterior once trained. In the case of GAN, the training data, the choice of loss functions and architecture parameters, and the upfront time spent in training yield an implicitly-built prior estimator in the form of trained network weights - allowing for near-instantaneous inference/reconstruction after training. The training process involves finding the optimal hyperparameters for both generator and discriminator, which requires high-performance computational resources such as modern GPUs. Although training a GAN can be challenging, one can save the trained generator and reuse it to generate an unlimited number of synthetic microstructures swiftly. This is an essential advantage of GANs over the classical stochastic methods. For example, the time required for training our GAN on $128^2$ meta-igneous images was about 3 h using two 24 GB NVIDIA Quadro P6000 GPUs. However, after training, it only needed 4 seconds to reconstruct 128 images, whereas the reconstruction time of the same number of images via SA was about 2 h on a system with 24 CPUs (Intel Xeon Gold 6136, 3 GHz), showing an acceleration of 1800 times.

### Reconstruction analysis of GANs

**Figure 9:**
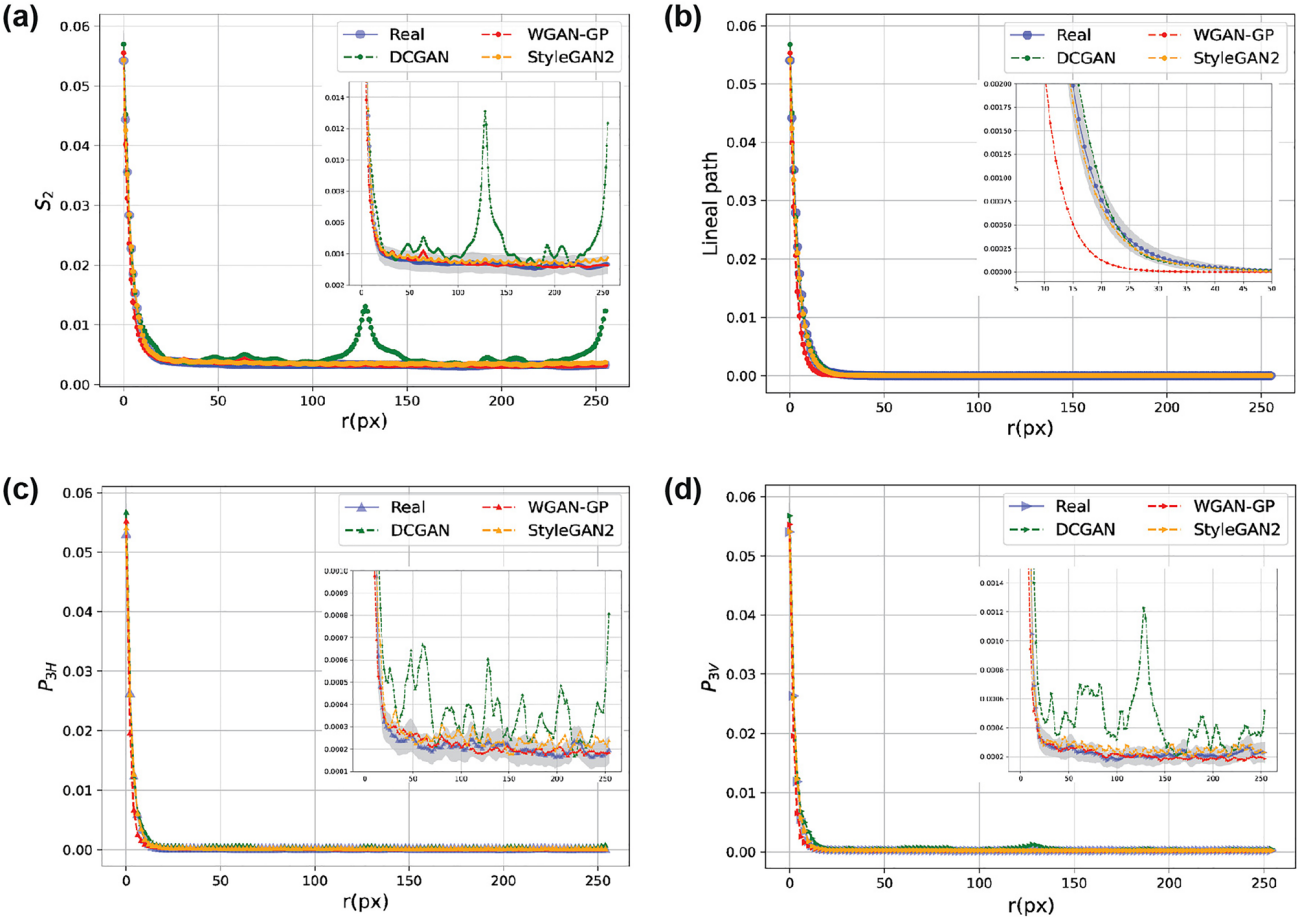
Quantification of original and reconstructed meta-igneous microstructures via different GANs. $P_4$ and $P_6$ are not presented due to insignificant amounts of information.

Figure 9 illustrates the quantification of original and reconstructed meta-igneous microstructures via SMD curves ($P_4$ and $P_6$ are not shown as they contain little information i.e., they become zero at small *r*). The strong correlations observed in $S_2$ curves at $r\approx 128$ and $r\approx 256$ reveal that some patterns are spatially repeated in the synthetic images by DCGAN, which is also illustrated by red arrows in Fig. 6c. Particularly, the peak at $r\approx 256$ shows that more pores are generated at the edge of the image. However, in agreement with visual inspection (Fig. 6), there is a good match between the $S_2$ curves at small ranges in all reconstructions, showing that the pore size and pore shape have been captured by all GANs. Despite the similar pore size, the lineal path curves indicate that WGAN-GP underestimates the connectivity i.e., there are black pixels within the pores, making it less probable that a random line lies entirely in the white phase. This artefact is known as the checkerboard pattern and can be observed by zooming in Fig. 6e. This was not unexpected as it might happen when the generator consists of transpose convolution layers as in WGAN-GP. Apart from the connectivity, other curves confirm the good match between WGAN-GP and StyleGAN2-ADA with original structures in the sample while DCGAN curves exceed the 95 percent confidence level at multiple ranges.

**Figure 10:**
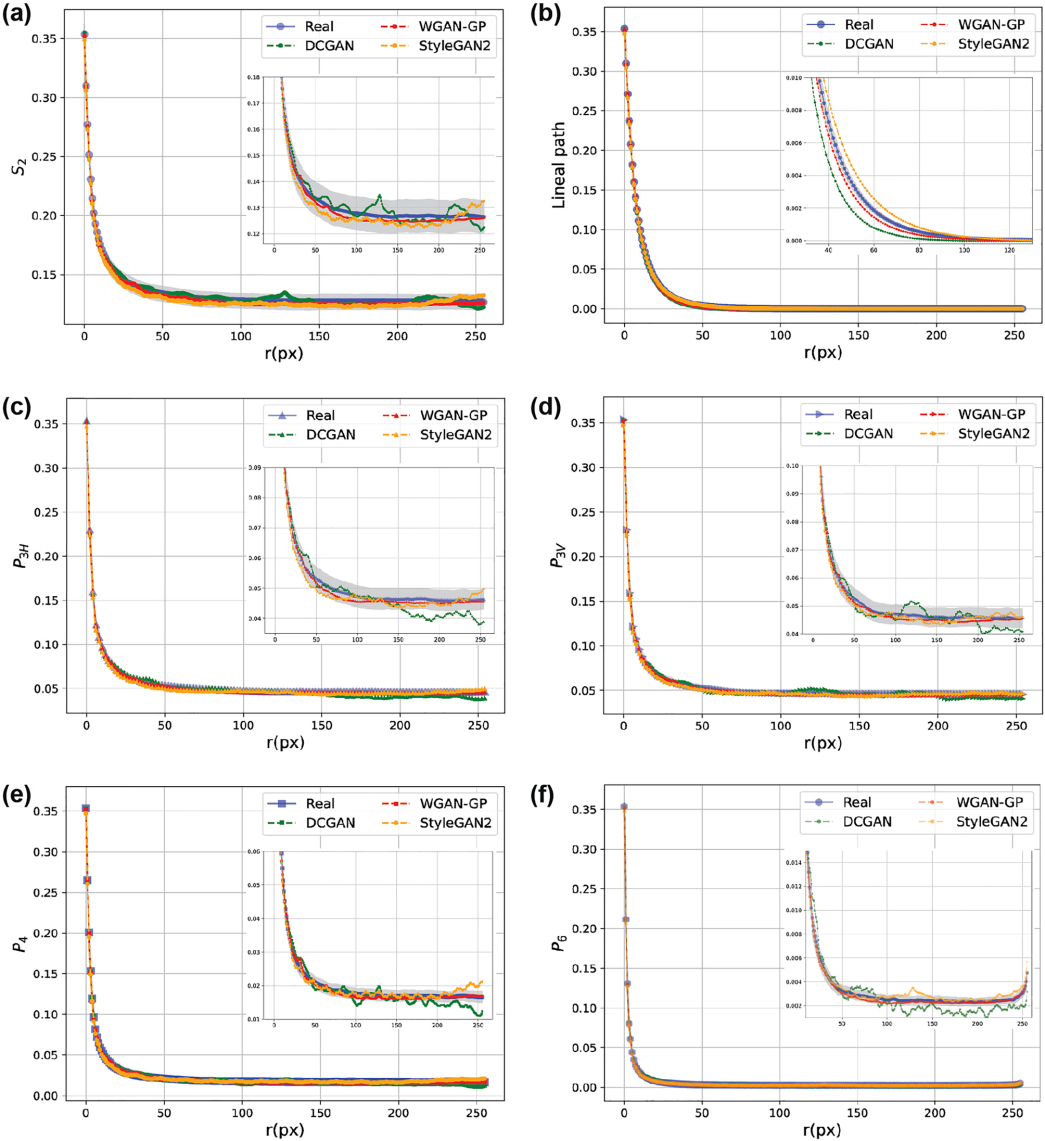
Characterising original and reconstructed serpentinite = − microstructures using different GANs. 95 percent confidence levels around the average values of real microstructure are shown by grey shadow.

In the case of serpentinite in Fig. 10, the average SMDs obtained from WGAN-GP and StyleGAN2-ADA are within the confidence bounds of the original ones, while DCGAN curves go off the confidence bounds at high-order correlations. Here, we can see that the DCGAN underestimates the linear connectivity of fractures while other GANs better match the lineal-path curves (Fig. 10b), consistent with Fig. 6 and the MSE errors presented in Table S4. However, an increased correlation is also observed in StyleGAN2-ADA curves (orange curves) at large ranges showing that more fractures (or in general white pixels) are generated at the edges of images than the original ones (see Fig. S1g). As mentioned before, mode collapse is another common failure case in DCGAN due to the BCE loss function (Eq. 5), and it happens when the generator collapses to a set of fixed or very similar outputs. Figure S1c shows 64 random images generated using the trained DCGAN. It can be seen that all the images are very similar, which is also apparent from Fig. S1d showing that the $S_2$ curves of reconstructed images via DCGAN are very close to each other. On the other hand, we can see that $S_2$ curves from WGAN-GP and StyleGAN2-ADA cover the whole range of real image correlations, indicating that these two models are able to generate not only high-fidelity but also diverse synthetic images. Table S4 also reports the MSE errors between original and reconstructed SMD curves, which confirms the superior performance of StyleGAN2-ADA over other variants.

In terms of computational time, it took about 15 and 33 h for DCGAN, and WGAN-GP to converge and give the best model on serpentinite images of resolution 512, respectively. Although the same learning rate ($1e^{-4}$) was used in both models, a longer training time was expected in WGAN-GP because *D* was updated 5 times for each *G* update in practice. Furthermore, the gradient penalty term used in the loss function requires one more forward and backward propagation. While training with a larger learning rate makes the training faster, our experiments show that WGAN-GP is sensitive to learning rate and becomes unstable at larger values. On the other hand, the training time for StyleGAN2-ADA was more than 80 h which stems from additional layers in mapping and synthesis of the networks (see^42^ for more details). Another reason is that more regularization terms were used in StyleGAN2-ADA involving $R_1$ regularization for *D* and path length regularization for *G*. The latter encourages a smoother *G* which means that a specific step size in *w* space results in a fixed change in the generated images. Another advantage of StyleGAN2-ADA over WGAN-GP was that we could successfully train it with images of higher resolution (1024) which was not possible in the case of other variants with our computational resources. This is because of skip connection and residual architecture used in *G* and *D*, respectively. Finally, the adaptive discriminator augmentation (ADA) pipeline in which allows training with limited data. For example, in the case of serpentinite, we trained StyleGAN2-ADA with 5600 real images without the problem of overfitting (which is common when training images are not sufficient) while 9100 images were used for training DCGAN and WGAN-GP.

## Conclusions and outlook

We investigated the use of three variants of GANs (DCGAN, WGAN-GP, and StyleGAN2-ADA) to reconstruct two-dimensional microstructures of natural rocks. We evaluate and compare their performance in retrieving highly-complex geometries accurately by quantifying higher-order statistical correlation functions. As inputs, we used electron microscopic images of two heterogeneous systems. The first sample is from an altered igneous rock with a mostly isolated but oriented pore network generated during fluid-driven mineral replacement. The second sample is from a serpentinised peridotite characterised by a complex and connected fracture network containing different geometrical patterns. Our results show that GANs are capable of capturing and reconstructing these topologically complex microstructures without prior statistical information - provided one takes steps to ensure training stability and to perform quality control after inference with the use of high-order descriptors.

Overall, for the first time, we carry out a comprehensive analysis of high-order SMDs for comparing the results of different GANs showing the strength of these tools in both training and inference time. One of the major challenges in training GANs is that, in contrast to supervised learning, the value function (Eq. 5) is not particularly useful to stop training. Our findings show that the proposed SDMs can be computed for a batch of images during training and be monitored for evaluating the accuracy of GANs in capturing spatial patterns and detecting failure cases and artefacts. Computing each SMD can be perceived as encoding an image into a 1D vector (*r* is the index of pixels and remains unchanged for the same image size). Then, a distance metric (e.g., MSE) between vectors of original and synthetic images can be calculated and used as criteria to stop training or save generator’s weights ($\theta ^{(G)}$). While our images were binary, this approach can also be utilized in multiphase systems. In this case, correlation functions should be computed for each phase to be compared with those in real microstructures. The drawback of incorporating descriptors for evaluation during the training is that it makes the training time longer, especially for images larger than 512 pixels. However, one can start such an evaluation when training (generator and discriminator losses) stabilizes, and perform it hierarchically i.e., calculating higher-order correlations only if there is a good match between lower-order curves.

Also, we showed that the proposed descriptors can be employed in inference time to not only validate GANs’ performance quantitatively but also interpret the synthetic microstructures. In other words, the proposed SMDs show not only *how much* but also *how* generated images differ from the original ones. The $S_2$ and Minkowski functionals are traditional metrics used by previous works to validate GANs. We showed and discussed the inadequacy of $S_2$ (Figs. 3 and 4) in the case of complex microstructures, illustrating that two microstructures can have almost identical $S_2$ yet be structurally and topologically different. Although Minkowski functionals are useful metrics in three dimensions for calculating morphological properties, the properties that can be derived from 2D images are area, boundary length, and 2D Euler connectivity. Our proposed statistical tools, however, provide more information to evaluate reconstructions more precisely. Evaluation of these higher-order spatial correlation functions enables us to validate that different geometrical patterns (i.e., two-point, horizontal and vertical triangle, square, and hexagon patterns) with all possible edge lengths in generated microstructures match the original patterns. However, the highest order we should compute depends on the complexity of the microstructure. For example, while there is not much spatial information in $P_4$ and $P_6$ in the case of the meta-igneous sample (i.e., these curves drop quickly to zero at small ranges), complex fracture networks in the serpentinite contain such patterns, making it necessary to take account of these higher-order statistics. Moreover, as average polytope functions are calculated, a bump at a certain distance in the average curve shows that a correlation exists at that distance in the original microstructure which should be captured by the reconstruction method as much as possible. On the other hand, we should avoid generating repeating patterns if they are not present in the real microstructure (Fig. 9). It should be mentioned that different metrics such as frechét inception distance (FID) and precision and recall (PR)^42^are common in computer vision for quality assessment. However, they are not interpretable as SMDs, and usually need a large dataset to compare the similarity between two distributions (i.e., $P_{data}$ and $P_{model}$) in high dimensions. Hence, they only show the general performance of the model to match the real data and do not ensure that each reconstructed image has the same structural and topological properties as your real images.

Last, based on the SMDs, we propose a novel methodology for determining the representative image size which is one of the first steps that should be taken in the image reconstruction problems. The representative image size determines the size of the training images, which in turn, is an important factor in selecting the GAN’s architecture and estimating the required computational resources. Our method is more comprehensive than the previous approaches - in terms of how it adapts to the properties of samples in question - because it considers the structural and morphological information captured by the selected SMD(s) used for the analysis.

The success of applying GANs for image reconstruction in recent years has resulted in increased interest and the emergence of new variants of GAN with new capabilities that can be easily incorporated due to the flexibility of GANs. Examples are Slice-GAN^35,37^, BicycleGAN^36^, and slice-to-pore GAN^38^, which all have been developed for 2D to 3D image reconstruction where 2D images of orthogonal planes are used to generate synthetic but statistically equivalent 3D microstructures. This type of GANs receives increased attention because 2D images are easier and more affordable to acquire and usually have higher resolution and a larger field-of-view (FoV). Moreover, patch-based GANs have been introduced to improve the reconstruction quality and the control of the local and global features. Chun et al.^29^ implemented a patch-based DCGAN for 2D reconstruction of heterogeneous energetic materials, and showed that it is possible to better control the micromorphology of reconstructions by introducing two input vectors by which one can manipulate the local stochasticity and global morphology of microstructure. This work was then expanded by^34^ by adding an actor-critic (AC) reinforcement learning to 3D DCGAN for generating microstructure with user-defined properties.

Despite high-quality image generation, much less is known about the properties of the latent space (z-space), for example, how image attributes are formed and organised in the latent space of a well-trained GAN, and the correlation between these attributes. Also, there is still uncertainty about how GANs can link the latent space to image semantic space and how the latent space can be interpreted and used for image manipulation^63^. The reason is that the generator in GANs is not trained to be invertible, i.e., a two-way mapping between the dataset (image space) and the latent space is not established during adversarial training. Instead, GANs learn to produce high-quality synthetic images indirectly by optimising the generator’s weights to imitate the original dataset according to feedback from discriminator. Learning such mapping between latent and image space allows us to explore the latent space and to manipulate realistically, edit, and combine the features in the generated image. Several studies have attempted to address this challenge by using inverse mapping, i.e., from image space to latent space. More information about such methods, known as GAN inversion, can be found in the comprehensive survey by^64^. Other approaches focus on coupling GANs with other generative models, such as variational autoencoder^65^. Employing such methods can be a fruitful area for future works to investigate how higher-order information (such as polytope functions) are encoded by GAN in latent space and intermediate semantic space, and how we establish a mapping between this information and macroscopic properties of rock.

We show that coupling SMDs, initially developed in material science, with deep learning equips us with a powerful set of tools to tackle wide-ranging geoscience research questions where the quantification of morphology-dependent rock properties is critical. This includes the generation of a wide library of digital samples from real samples to assess the statistical significance and/or variability of rock transport properties, e.g., in the presence of multi-phase flow. Moreover, our work enables new upscaling schemes, i.e., to generate representative domains from high-resolution images of small complex samples, and hence to couple microstructures to macroscopic phenomena as well as to enable the reconstruction of 3D microstructures when only 2D images are available. In both applications, higher-order characterisation is essential to ensure that the artificially-generated images share the same structural and topological properties. A particular example based on the rock microstructures studied here is the application of deep-learning-based image reconstruction to reactive fluid flow in which, e.g., a connected pore network exists at the time of reaction. However, most pores are isolated after the reaction ceases^2,66^, as observed in the meta-igneous rock system. In such a situation, GAN inversion methods may realistically reconnect the isolated pores back to the time of reaction. In our future work, we try to couple the StyleGAN2 inversion method with SMDs to capture transient processes and the microstructure evolution occurring within the solid Earth using time-resolved 3D imaging.

## Supplementary Information

Supplementary Information.

## Data Availability

Original segmented BSE images and the data to reproduce the figures as well as python codes are available at Utrecht University Yoda data repository accessible via: https://public.yoda.uu.nl/geo/UU01/ACSDR4.html. Python codes are also accessible via https://github.com/hamediut/GeoWGAN-GP.
