# Supplementary material for: Quantifying microstructures of earth materials using higher-order spatial correlations and deep generative adversarial networks
Source: Sci Rep. 2023 Jan 31;13:1805. doi: 10.1038/s41598-023-28970-w (PMC9889385; doi:10.1038/s41598-023-28970-w)
Supplement: Supplementary file 1 — Supplementary Information. [file 41598_2023_28970_MOESM1_ESM.pdf]

Supplementary Information for "Quantifying microstructures of earth materials using higher-order spatial correlations and deep generative adversarial networks"

Hamed Amiri[1,*], Ivan Vasconcelos[1], Yang Jiao[2], Pei-En Chen[3], and Oliver Plümper[1]

[1]Department of Earth Sciences, Utrecht University, Utrecht, the Netherlands
[2]Materials Science and Engineering, Arizona State University, Tempe, U.S.A.
[3]Mechanical and Aerospace Engineering, Arizona State University, Tempe, U.S.A.
[*]h.amiri@uu.nl

ABSTRACT

Table S1. presents the architectures used in the WGAN-GP for the reconstruction of images of resolution 128. A similar architecture, but with extra layers, is needed in both generator and discriminator for larger images. This architecture differs from that of the DCGAN in which batch normalisation layers are applied in the Discriminator. Note that no batch normalisation should be applied in the discriminator's layers of WGAN-GP, otherwise it does not improve the stability. This is critical in the WGAN-GP as the norm of the discriminator's gradient is penalized according to each image instead of the entire batch[1]. A convolution layer is also added before the last layer in the generator to alleviate the problem of checkerboard pattern artefacts created by the uneven overlap in the transpose convolution layers[2]. In the generator, however, batch normalisation is followed at each layer by applying a rectified linear unit (ReLU) or leaky ReLU as activation functions[3,4]. Our investigation indicates better results are obtained when no activation function is applied in the discriminator's last layer.

Table S2 provides the hyperparameters for training the WGAN-GP in both case studies. The same hyperparameters were used for DCGAN, except using a batch size of 32. This reason is that extra batch normalisation layers in DCGAN's discriminator increase the model volume and number of trainable parameters such that we had to decrease the batch size due to the limited GPU memory. Network weights were first randomly initialised and then were updated at each iteration by the Adam optimiser[5] using the reported learning rate and momenta. Discriminator repeats are the number of times the discriminator's weights were updated for each generator update. The λ is the coefficient of the regularization term in the WGAN-GP loss function whose role is to penalize the gradient norm of the critic (with respect to its input) from one. The larger the λ, the smoother the training evolution, making the WGAN-GP more stable. Our experiments (on the meta-igneous sample of resolution 128) show that the combination of λ and the learning rate is important. For example, for a small and fixed learning rate of $1e^{-4}$, critic loss (as well as generator loss) shows more fluctuations at $\lambda < 10$. On the other hand, stabilised training was observed for $\lambda \geqslant 10$, and both losses steadily converged. However, for a higher learning rate of $1e^{-3}$, the losses oscillated heavily even with a large value of $\lambda = 100$, giving unrealistic results. Therefore, findings of this study suggest that a learning rate of $1e^{-4}$ and $\lambda \geqslant 10$ should be used in WGAN-GP for obtaining stable training and high-quality reconstructions.

Table S3 reports the mean square errors (MSEs) between spatial-correlation functions derived from original and reconstructed microstructures of size 128 using SA and WGAN-GP methods. It can be seen that the MSE associated with WGAN-GP is two to three orders of magnitude less than SA, except for the two-point correlation function. Similarly, Table S4 compares the accuracy of three variants of GANs implemented on representative size of 512. In general, these quantitative results show the better performance of StyleGAN2-ADA over WGAN-GP which, in turn, performs better than DCGAN in most cases.

Fig. S1 illustrate 64 random reconstructions of serpentinite sample via each GAN, as well as corresponding S_2 curves to show how SMDs can be useful in mode collapse detection. The narrow range of S_2 curves in DCGAN (Fig. S1d) means that very similar patterns are reproduced by the DCGAN. In contrast, it can be seen that both WGAN-GP and StyleGAN2-ADA cover the whole range of diversity quantified by S_2 curves original microstructure.

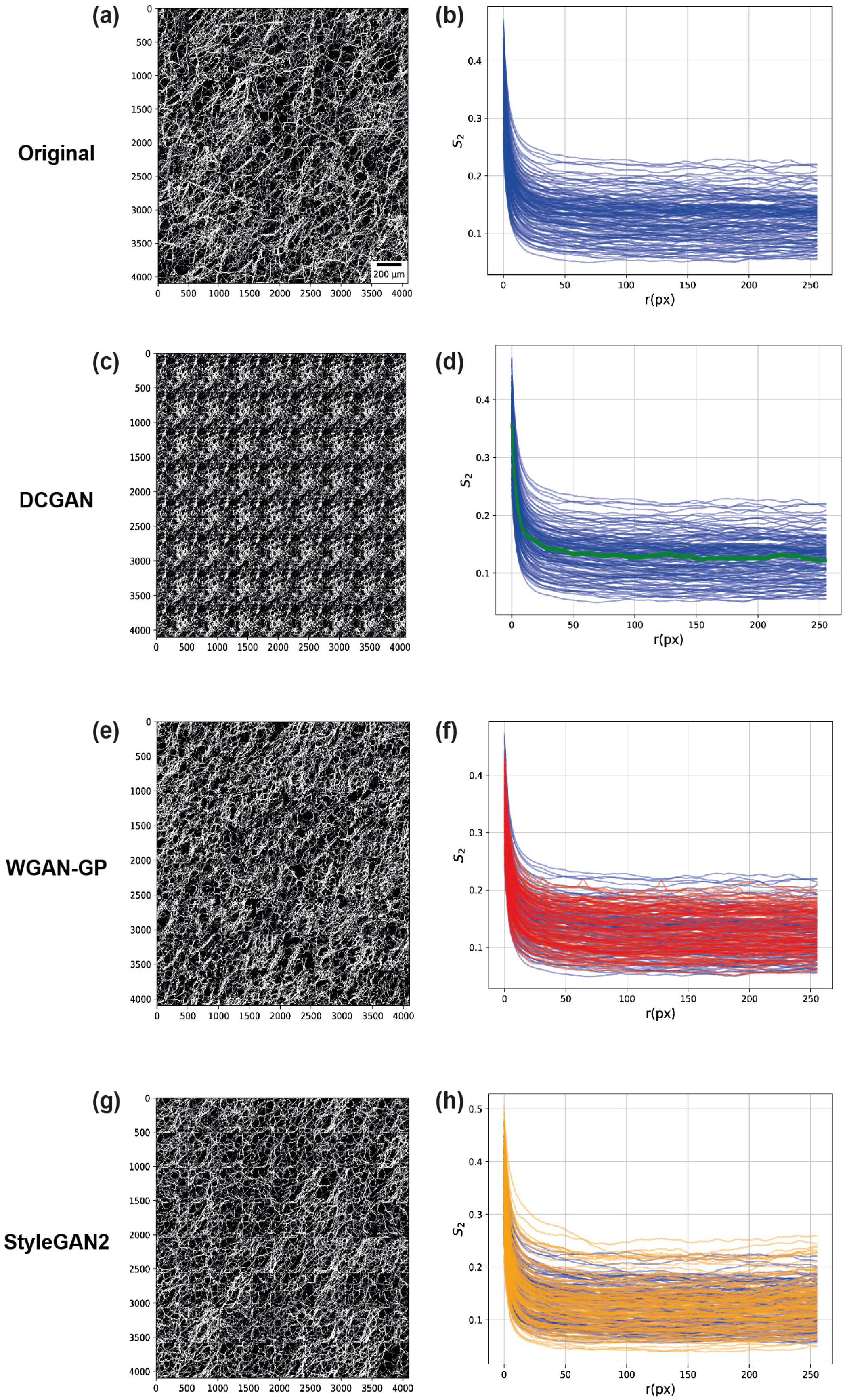

Figure 1. Diversity of images generated by each GAN. Each image in the first column shows 64 random images of resolution 512 stitched together. S_2 curves of images are shown in the second column for the original (blue), DCGAN (green), WGAN-GP (red), and StyleGAN2-ADA (orange).

Table S 1. Generator and discriminator architecture in this study.

Layer	Type	Filters	Kernel	Stride	Padding	Batch	Activation
**Generator**							
1	ConvTrans2D	1024	4×4	1	0	Yes	ReLU
2	ConvTrans2D	512	4×4	2	1	Yes	ReLU
3	ConvTrans2D	256	4×4	2	1	Yes	ReLU
4	ConvTrans2D	128	4×4	2	1	Yes	ReLU
5	ConvTrans2D	64	4×4	2	1	Yes	ReLU
6	Conv2D	64	1×1	1	0	Yes	ReLU
7	ConvTrans2D	1	4×4	2	1	No	Tanh
**Discriminator**							
1	Conv2D	64	4×4	2	1	No	LeakyReLU
2	Conv2D	128	4×4	2	1	No	LeakyReLU
3	Conv2D	256	4×4	2	1	No	LeakyReLU
4	Conv2D	512	4×4	2	1	No	LeakyReLU
5	Conv2D	1024	4×4	2	1	No	LeakyReLU
6	Conv2D	1	4×4	1	0	No	None

Table S 2. Training parameters used in this study.

Batch size	128
Noise vector (z) dimension	512
Generator filters	64
Discriminator filters	64
Learning rate (α)	1^{-4}
Momenta(β_1, β_2)	(0.5, 0.999)
Discriminator repeats	5
Coefficient(λ)	10

References

1. Gulrajani, I., Ahmed, F., Arjovsky, M., Dumoulin, V. & Courville, A. C. Improved training of wasserstein gans. *Adv. neural information processing systems* **30** (2017).

2. Odena, A., Dumoulin, V. & Olah, C. Deconvolution and checkerboard artifacts. *Distill* **1**, e3 (2016).

3. Maas, A. L., Hannun, A. Y., Ng, A. Y. *et al.* Rectifier nonlinearities improve neural network acoustic models. In *Proc. icml*, vol. 30, 3 (Citeseer, 2013).

4. Shang, W., Sohn, K., Almeida, D. & Lee, H. Understanding and improving convolutional neural networks via concatenated rectified linear units. In *international conference on machine learning*, 2217–2225 (PMLR, 2016).

5. Kingma, D. P. & Ba, J. Adam: A method for stochastic optimization. *arXiv preprint arXiv:1412.6980* (2014).

Table S 3. The assessment of image reconstruction quality using SA and our WGAN-GP. The values are mean square errors (MSEs) calculated between correlation functions of original and reconstructed images of resolution 128 as shown in Fig. 4.

Correlation functions	Meta-igneous		Serpentinite	
	SA	WGAN-GP	SA	WGAN-GP
S_2	8.26×10^{-6}	2.16×10^{-5}	5.94×10^{-5}	4.42×10^{-5}
P_{3H}	5.23×10^{-5}	2.96×10^{-7}	1×10^{-3}	2.13×10^{-6}
P_{3V}	7.69×10^{-5}	4.72×10^{-7}	1.27×10^{-3}	3.78×10^{-6}
P_4	6×10^{-5}	1.61×10^{-6}	1.33×10^{-3}	5.74×10^{-6}
P_6	2.98×10^{-5}	2.56×10^{-7}	1.19×10^{-3}	1.44×10^{-6}

Table S 4. Comparing reconstruction performance of GANs used in this study. The values are MSEs between correlation functions of original and each GAN model, obtained from meta-igneous (Fig. 9) and serpentinite (Fig. 10) samples.

Correlation functions	Meta-igneous			Serpentinite		
	DCGAN	WGAN-GP	StyleGAN2	DCGAN	WGAN-GP	StyleGAN2
S_2	4.19×10^{-6}	6.15×10^{-7}	6.87×10^{-8}	6.82×10^{-6}	2.93×10^{-6}	6.56×10^{-6}
P_{3H}	1.64×10^{-7}	7.91×10^{-7}	1.01×10^{-8}	1.45×10^{-5}	1.68×10^{-6}	3×10^{-6}
P_{3V}	2.11×10^{-7}	7.04×10^{-7}	4.31×10^{-9}	8.14×10^{-6}	1.57×10^{-6}	3.76×10^{-6}
P_4	6.55×10^{-8}	7.38×10^{-7}	3.73×10^{-8}	4.46×10^{-6}	7.15×10^{-7}	3.07×10^{-6}
P_6	3.79×10^{-8}	6.27×10^{-7}	1.9×10^{-8}	7×10^{-7}	1.42×10^{-7}	2.83×10^{-7}
L	5.29×10^{-8}	1.5×10^{-6}	4.4×10^{-8}	1.91×10^{-6}	1.43×10^{-7}	4.2×10^{-7}
